# Associations Between Insulin Resistance, Free Fatty Acids, and Oocyte Quality in Polycystic Ovary Syndrome During In Vitro Fertilization

**DOI:** 10.1210/jc.2013-3942

**Published:** 2014-04-02

**Authors:** Zhihong Niu, Nan Lin, Ruihuan Gu, Yijuan Sun, Yun Feng

**Affiliations:** IVF Unit, Department of Obstetrics and Gynecology, Ruijin Hospital Affiliated to Shanghai Jiaotong University, Shanghai 200025, China

## Abstract

**Context:**

Both polycystic ovary syndrome (PCOS) and obesity are associated with specific reproductive health complications, including lower oocyte quality and clinical pregnancy rates in assisted conception cycles, which may be a result of metabolism-induced changes in the oocyte through the microenvironment of follicular fluid. Free fatty acids (FFAs) are important biomedical indicators of abnormal lipid metabolism and have pronounced effects on cells, leading to changes in metabolism, cell growth, and differentiation

**Objective:**

Our objective was to determine the effect of FFA metabolism in plasma and follicular fluid on oocyte quality in the women with PCOS undergoing in vitro fertilization.

**Design and Setting:**

Ninety-three women undergoing in vitro fertilization treatment, including 55 with PCOS and 38 age-matched controls, were recruited. PCOS patients were divided into obese and nonobese subgroups on the basis of their body mass index.

**Main Outcome Measures:**

Embryo quality was morphologically assessed, and serum sex hormone and insulin levels were measured. FFAs in plasma and follicular fluid were measured using gas chromatography-mass spectrometry.

**Results:**

PCOS was found to be associated with significantly higher LH/FSH, total T, free androgen index (FAI), and lower SHBG levels, independent of obesity(*P* < .05). Obese women with PCOS had a significantly higher total T level, FAI, fasting insulin, insulin resistance index as determined by homeostasis model assessment for insulin resistance, and lower SHBG levels than the nonobese women with PCOS (*P* < .05). The embryo fragmentation score was significantly positively correlated with the oleic acid concentration in all PCOS patients (r = 0.22, *P* = .04, for nonobese patients and r = 0.25, *P* = .03, for obese patients).

**Conclusions:**

Our findings clearly demonstrated that PCOS is associated with significantly higher FAI and insulin resistance levels and decreased plasma SHBG levels, independent of body mass index. Obese PCOS patients had higher palmitoleic acid and oleic acid levels in both the plasma and follicular fluid than did the control subject and nonobese PCOS patients. Our results indicated that developmental competence is associated with oleic and stearic acid concentrations, which may contribute to the poor pregnancy outcomes in patients with PCOS.

Polycystic ovary syndrome (PCOS) is the most common endocrine disorder among women of reproductive age, with a prevalence of 5%–10% (1, 2). Hyperandrogenism, oligomenorrhea, chronic anovulation, and hyperinsulinemia are common clinical manifestations of PCOS. The systemic changes that occur in PCOS reflect not only ovarian function but also the changes in whole-body metabolism, including obesity, insulin resistance (IR), and diabetes. Of these, IR is associated with an increased risk of impaired glucose tolerance or manifest type 2 diabetes, lipid disturbances, and cardiovascular disease (3–5). Accordingly, several studies have reported an increased prevalence of impaired glucose tolerance, type 2 diabetes, and dyslipidemia in women with PCOS (6–9). Obesity acts as a major trigger in the occurrence of these disorders, which are associated with IR, in women with PCOS. Other studies have reported that both PCOS and obesity are associated with specific reproductive health complications, including lower clinical pregnancy rates in assisted conception cycles (10–12). These outcomes may be a result of metabolism-induced changes in the oocyte through the microenvironment of follicular fluid.

Studies on animal models of maternal diabetes provide evidence that abnormal maternal physiology affects oocytes and results in abnormal pregnancy outcomes. Oocytes from diabetic, insulin-resistant, and obese mice show delayed maturation, smaller size, and increased granulosa cell apoptosis (13–15). These findings are linked to adverse embryonic and fetal outcomes, including delayed embryonic development, growth restriction, anatomical defects, and smaller fetuses (16, 17). Recent studies indicate that free fatty acids (FFAs) are important biomedical indicators of abnormal lipid metabolism and have pronounced effects on gene expression, leading to changes in metabolism, cell growth, and differentiation (18, 19). It was indicated that the fatty acid composition of oocytes and their environment influence developmental competence (20, 21) .

Therefore, the present study aimed to provide insights into the effects of FFA metabolism in the plasma and follicular fluid on oocyte quality in women with PCOS undergoing in vitro fertilization (IVF). We also examined the relationship between insulin sensitivity and FFA metabolism in the follicular fluid of PCOS patients.

## Materials and Methods

### Patients

Ninety-three women undergoing IVF treatment at the Reproductive Center of Ruijin Hospital affiliated to Shanghai Jiaotong University Medical School were recruited to this study. Of these, 55 women had PCOS and 63 were age- and BMI-matched controls. According to the 2003 Rotterdam criteria, the presence of two or more of the following signs were considered to be diagnostic of PCOS: oligoovulation and/or anovulation, clinical and/or biochemical signs of hyperandrogenism, and polycystic ovaries after exclusion of other etiologies (such as congenital adrenal hyperplasia, androgen secreting tumors, Cushing syndrome, 21-hydroxylase deficient nonclassic adrenal hyperplasia, androgenic/anabolic drug use or abuse, thyroid dysfunction, hyperprolactinemia, type 2 diabetes mellitus, and cardiovascular disease). Women who had received any hormonal treatment or insulin-lowering agent within the last 3 months were excluded from the study. Patients were divided into four groups: 1) lean (BMI 18.5–24.9 kg/m^2^), non-PCOS; 2) lean PCOS; 3) obese (BMI > 30 kg/m^2^), non-PCOS; and 4) obese (BMI > 30 kg/m^2^), PCOS. The non-PCOS subjects were selected from women who visited the same reproductive center to receive IVF treatment owing to fallopian tube disorders.

Informed consent was obtained from all the patients before the IVF protocol, and the research protocol was approved by the institutional review board of our hospital.

### Stimulation protocols

Patients underwent regular ovarian stimulation and medical management in IVF center. In brief, a GnRH antagonist protocol was applied for ovarian stimulation. Each patient received individual doses of gonadotropins, FSH, and/or human menopausal gonadotropin, starting from day 2 or 3 of her menstrual cycle. They then received sc injections of 0.125 mg of the GnRH antagonist cetrorelix acetate (Cetrotide; Merck Serono) from day 6 of stimulation. Stimulation was monitored using estradiol concentrations, together with ultrasound measurements of follicle numbers and diameters. Ovulation was induced using human chorionic gonadotropin when the leading follicle reached 18–20 mm in diameter.

### Sample collection

Baseline blood samples were collected between days 3 and 5 of the menstrual cycle in the control group and 3–5 days after a spontaneous bleeding episode in patients with PCOS after an overnight fast of 10–12 hours. In women with PCOS who did not have a spontaneous bleeding episode for 90 days, 60 mg progesterone was administered to induce a bleeding episode, and blood samples were collected afterward. Plasma was obtained from whole-blood samples collected in tubes containing ethylenediamine tetraacetic acid as the anticoagulant. The plasma samples were stored at −80°C until gas chromatography (GC) analysis.

Preovulatory ovarian follicular fluid was collected during transvaginal ultrasound-guided oocyte retrieval. Only follicular fluid samples that were found to be free of blood upon macroscopic analysis were retained for further analyses. Samples were obtained when the follicles measured 18–22 mm in diameter. The follicular fluid samples were collected in capped disposable polypropylene tubes, mixed with an equal volume of 0.5 M trichloroacetic acid, and centrifuged at 15 000 × *g* for 30 minutes. The supernatant was purified on a cation-exchange resin and used for subsequent GC analysis as described below.

### Assessment of embryo quality

**Figure 1.**
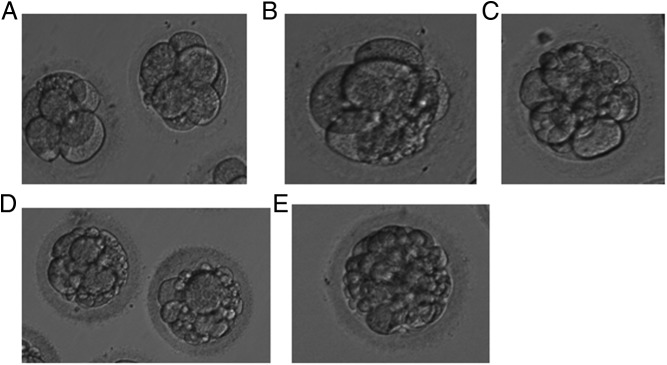
Embryo fragmentation scores.

Embryo morphology was evaluated on day 3 by assessing the embryo cell number and the percentage of fragmentation [embryo fragment score (EFS)]. EFS was graded on the basis of the percentage of fragmentation as follows: 5% or less fragmentation, score 4; 5%–10% fragmentation, score 3; 11%–25% fragmentation, score 2; 26%–50% fragmentation, score 1; and 51% or greater fragmentation, score 0 (Figure 1).

### Laboratory analysis

Serum sex hormones (FSH, LH, estradiol, and total T) and the serum insulin level were analyzed using commercially available kits from the Unicel DXI 800 Access immunoassay system (Beckman Coulter). The inter- and intraassay coefficients of variation were all less than 10%. SHBG was analyzed by an ELISA (USCNLIFE). Intra- and interassay variations were 5.2% and 7.5%, respectively. The concentrations of glucose and lipoproteins [total cholesterol, triglycerides, low density lipoprotein (LDL), and high density lipoprotein (HDL)] were measured using Unicel DXC 800 Synchron (Beckman Coulter). The inter- and intraassay coefficients of variation of LDL and HDL were 4.8%, 5.1% and 5.2% and 5.6%, respectively.

BMI was calculated by dividing the weight in kilograms by the square of the height in meters to assess obesity. The free androgen index (FAI) was calculated as the ratio of total T levels (nanomoles per liter) to SHBG levels (nanomoles per liter) × 100 (percentage). The IR index as determined by homeostasis model assessment (HOMA-IR) was calculated to estimate the insulin sensitivity index as fasting insulin (milliunits per milliliter) × fasting plasma glucose (millimoles per liter)/22.5, as described before (22). The inter- and intraassay coefficients of variation of insulin and glucose were 6.4% and 6.9% and 9.7% and 8.9%, respectively.

### GC/mass spectrometry (MS) analysis

GC-MS analysis was performed according to Agilent's specifications (23). Plasma aliquots and follicular fluid (100 μL) were thawed to 4°C. Each aliquot was supplemented with 20 μL of internal standard solution (1 μg/μL succinic-d4 acid; Sigma Aldrich). Proteins were precipitated by adding 900 μL of cold methanol/water [8:1 (vol/vol)], followed by ultrasonication for 4 minutes and vortex mixing for 10 seconds. After centrifugation for 10 minutes (19 000 × *g*, 4°C), two replicates of 200 μL each of the supernatants were transferred to a GC autosampler vial, and 20 μL myristic-d27 acid (Sigma Aldrich) was added to each aliquot. The samples were then used as internal standards for retention time lock (RTL system provided in ChemStation Software; Agilent Technologies). Finally, the mixture was lyophilized overnight at room temperature. To prevent batch effect, all the assays were conducted in a random and double-blind manner. Then 200 μL of the reference standard was transferred to 2.0-mL Eppendorf tubes, derivatized with the procedure illustrated above, and prepared for GC/MS analysis as described below.

Each 1-μL aliquot of the derivatized solution or reference standard was injected in splitless mode into an Agilent 7890A GC system equipped with an Agilent DB-5MS capillary column (30 mm × 0.25 mm internal diameter × 0.25 μm film thickness) and an Agilent 5975C Series autosampler (Agilent Technologies). Helium was used as the carrier gas at a constant flow rate of 1.0 mL/min. The temperatures of the inlet, transfer line, ion source, and quadrupole were maintained at 270°C, 260°C, 200°C, and 150°C, respectively. The temperature protocol was set as follows: isothermal heating at 80°C for 2 minutes, followed by oven temperature ramps at the rate of 10°C/min to 180°C, 5°C/min to 240°C, and 10°C/min to 26°C, and a final maintenance at 260°C for 8 minutes. Data acquisition was achieved using MS in electron impact mode and full-scan monitoring mode over a mass to charge ratio range of 30–600 with an acquisition rate of 20 spectra/sec. The solvent delay time was set at 5 minutes.

### Data processing for GC/MS and pattern recognition

After GC/MS analysis, each sample or reference standard was represented by a GC/MS total ion current chromatogram. Among the detected peaks, a multidimensional vector was manually constructed to characterize the compounds. Each vector was normalized to the total sum of vector intensity, thereby partially accounting for the different concentrations due to the different sample sizes used. The peaks occurring due to column bleed and derivatization reagent were removed. Only peaks that were consistently detected in at least 80% of the samples were included in the analysis. All the detected peaks were identified by the National Institute of Standards and Technology mass spectral library (Wiley registry, 2008 edition) and customized reference mass spectral libraries (23, 24).

The mass spectra obtained were manually investigated, and molecules with matching probability of greater than 80% were considered. Within each sample, the retention time and mass to charge ratio data pairs were used as the identifier for each peak, and the ion intensities for each detected peak were then normalized to the sum of the peak intensities in that sample. To account for any differences in concentration between samples, all data were normalized to a total value of 100.

### Data analysis

Results are presented as means ± SD or SEM. Continuous clinical variables were analyzed by two-way ANOVA, and categorical variables were analyzed by the χ^2^ test. GC-MS variables were rank transformed before being subjected to two-way ANOVA because most of them did not follow a normal distribution. Primary analyses were performed using the Statistical Package for the Social Sciences version 14.0 (SPSS Inc), with a value of *P* < .05 considered to be statistically significant.

## Results

In the total study population, women with and without PCOS were comparable in age (Table 1). Of the 55 women with PCOS, 25 had a BMI within the normal range (average, 22.9 ± 3.1 kg/m^2^), whereas 30 women had a BMI greater than 30 kg/m^2^ (average, 32.4 ± 2.4 kg/m^2^). PCOS was associated with significantly higher LH to FSH ratio, total T, FAI, and lower SHBG levels, independent of obesity. Women with PCOS also had significantly higher HOMA-IR ratio independent of obesity. Among the women with PCOS, obese women had significantly higher total T, FAI, fasting insulin, HOMA-IR, and lower SHBG levels than those with normal BMI. Furthermore, obese patients with PCOS showed significantly higher fasting insulin, triglycerides, and LDL levels and lower HDL levels than the control group. In non-PCOS patients, obese women had higher fasting insulin, HOMA-IR, LDL, and lower SHBG level than lean women.

**Table 1.** Baseline characteristics of the subjects

	Lean non-PCOS (n = 38)	Obese non-PCOS (n = 25)	Lean PCOS (n = 30)	Obese PCOS (n = 25)
Age, y	30.6 ± 3.5	30.9 ± 3.6	31.2 ± 3.7	30.9 ± 3.8
BMI, kg/m^2^	22.1 ± 2.8	32.9 ± 2.2^ab^	22.9 ± 3.1	32.4 ± 2.4^ab^
LH/FSH	0.4 ± 0.3	0.5 ± 0.3	0.8 ± 0.4^a,c^	0.8 ± 0.4^a,c^
Total T, nmol/L	1.53 ± 0.4	1.49 ± 0.6	1.75 ± 0.8	2.04 ± 0.8^a,b,c^
FAI, pmol/L	1.7 ± 0.4	1.8 ± 0.7	2.4 ± 0.7^a,c^	3.9 ± 1.2^a,b,c^
SHBG, nmol/L	97.5 ± 39	59.2 ± 32^a,b^	81.6 ± 29^a^	58.3 ± 45^a,b^
Fasting glucose, mmol/L	5.4 ± 0.4	5.5 ± 0.5	5.6 ± 0.5	5.7 ± 0.6
Fasting insulin, pmol/L	27 ± 13	52 ± 16^a,b^	36 ± 19	63 ± 32^a,b,c^
HOMA-IR	0.9 ± 0.5	2.0 ± 1.0^a,b^	1.4 ± 0.8^a^	2.4 ± 0.9^a,b,c^
TC, mmol/L	4.6 ± 0.2	4.7 ± 0.4	4.8 ± 0.2	5.1 ± 0.4
TG, mmol/L	1.2 ± 0.08	1.5 ± 0.12	1.5 ± 0.11	1.6 ± 0.17^a^
HDL, mmol/L	1.4 ± 0.05	1.3 ± 0.05	1.3 ± 0.03	1.1 ± 0.05^a^
LDL, mmol/L	2.5 ± 0.14	3.1 ± 0.11^a^	2.7 ± 0.09	3.2 ± 0.07^a^

Abbreviation: TG, triglyceride.

a Compared with lean non-PCOS (*P* < .05).

b Compared with lean PCOS (*P* < .05).

c Compared with obese non-PCOS (*P* < .05).

Table 2 summarizes the FFA levels in the plasma and follicular fluid derived from the GS-MS study of the study participants. The undecanoic acid (C11:0), nutmeg bean acid (C14:0), and stearic acid (C18:0) levels in the plasma and follicular fluid did not differ between the control subjects and PCOS patients. Both in serum and follicle fluid, the palmitoleic acid (C16:0) and oleic acid (C18:1n9cis) levels were higher in obese PCOS patients than other three groups. In addition, in lean PCOS patients, C18:1n9cis levels were higher than non-PCOS women, independent of obesity. From the data of follicular fluid, C16:0 levels were observed to be higher in obese women than lean women in the no-PCOS subgroup, but this difference did not exist in serum samples.

**Table 2.** Distributions of Fatty Acid in Serum and Follicular Fluid

	Lean Non-PCOS (n = 38)	Obese Non-PCOS (n = 25)	Lean PCOS (n = 30)	Obese PCOS (n = 25)
Serum				
C11:0, μg/mL	201.4 ± 112.8	197.3 ± 101.4	211.3 ± 107.6	223.5 ± 124.6
C14:0, μg/mL	81.3 ± 29.4	86.4 ± 31.5	85.6 ± 31.2	79.3 ± 29.2
C16:0, μg/mL	589.4 ± 120.3	614.3 ± 133.7	702.4 ± 131.7^a^	876.9 ± 146.5^a,b,c^
C18:0, μg/mL	241.6 ± 144.2	275.6 ± 153.8	238.5 ± 156.3	258.4 ± 179.0
C18:1n9cis, μg/mL	118.5 ± 27.6	154.2 ± 31.5^a^	198.4 ± 35.5^a,b^	269.7 ± 38.4^a,b,c^
C18:2n6cis, μg/mL	266.2 ± 67.8	299 ± 76.4	297.5 ± 88.3	354.2 ± 80.5^a,b,c^
Follicular fluid				
C11:0, μg/mL	183.6 ± 104.4	164.2 ± 107.5	176.4 ± 112.3	188.2 ± 118.0
C14:0, μg/mL	77.1 ± 26.7	80.5 ± 30.7	80.2 ± 29.2	83.6 ± 27.5
C16:0, μg/mL	434.2 ± 114.3	582.4 ± 127.9^a^	567.2 ± 121.0^a^	733.5 ± 152.2^a,b,c^
C18:0, μg/mL	230.7 ± 136.1	241.9 ± 148.0	227.1 ± 141.8	254.6 ± 161.7
C18:1n9cis, μg/mL	109.4 ± 22.4	121.6 ± 45.8	175.3 ± 44.2^a,b^	240 ± 62.8^a,b,c^
C18:2n6cis, μg/mL	218.9 ± 54.1	230.1 ± 58.6	220.3 ± 56.2	208.5 ± 60.0

a Compared with lean non-PCOS (*P* < .05).

b Compared with obese non-PCOS (*P* < .05).

c Compared with lean PCOS (*P* < .05).

As expected, the total FFA concentration in the follicular fluid and plasma were correlated in all groups (*P* < .05). Through Spearman correlation analysis, we also discovered a weak correlation between the total FFA levels in the follicular fluid and the plasma triglyceride levels among PCOS patients (*P* < .05). In lean PCOS patients, there was also a weak correlation between the total FFA levels in the follicular fluid and IR. No other significant correlations between the total FFAs in the follicular fluid and BMI, FAI, total cholesterol (TC), or IR were noted (Table 3).

**Table 3.** Correlation of Total FFAs in Follicular Fluid With BMI, FAI, IR, and Plasma Triglycerides

	Lean Non-PCOS	Obese Non-PCOS	Lean PCOS	Obese PCOS
	r	r	r	r
BMI	0.14	0.22	0.24	0.20
FAI	−0.18	−0.15	−0.11	−0.12
TC	0.15	0.31	0.24^a^	0.22
TG	0.11	0.18	0.17^b^	0.14^b^
IR	0.22	0.18^a^	0.11^b^	0.15^a^
Serum FFAs	0.47^b^	0.58^b^	0.42^b^	0.47^b^

Abbreviation: TG, triglyceride.

a *P* = .05.

b *P* < .05.

To further investigate the effects of FFA levels in the follicular fluid on oocyte quality, correlations between embryo fragmentation or blastomere score and specific FFA levels in the follicular fluid were measured (Table 4). The embryo fragmentation score was found to be significantly positively correlated with the follicular fluid oleic acid concentration in both nonobese (r = 0.22, *P* = .04) and obese PCOS patients (r = 0.25, *P* = .03). In the nonobese PCOS patients, no meaningful correlations were detected between embryo blastomere score and the levels of other specific FFAs in the follicular fluid. In obese PCOS patients; however, a significant negative correlation was observed between the blastomere score and stearic acid concentration in follicular fluid (r = −0.16, *P* = .04).

**Table 4.** Correlation between specific FFAs in follicular fluid and embryo quality in PCOS patients

	Nonobese				Obese			
	Embryo fragmentation		Blastomere score		Embryo fragmentation		Blastomere score	
	r	*P*	r	*P*	r	*P*	r	*P*
C11:0, μg/mL	0.07	0.17	0.13	0.07	0.02	0.09	−0.09	0.16
C14:0, μg/mL	0.19	0.11	0.08	0.11	0.13	0.12	0.11	0.14
C16:0, μg/mL	0.32	0.08	0.11	0.08	0.24	0.21	0.29	0.08
C18:0, μg/mL	−0.14	0.15	−0.29	0.06	−0.17	0.15	−0.16	0.04
C18:1n9cis, μg/mL	0.22	0.04	−0.23	0.06	0.25	0.03	−0.28	0.09
C18:2n6cis, μg/mL	−0.08	0.07	0.09	0.10	−0.13	0.08	0.15	0.18

## Discussion

The present study was performed to elucidate the characteristics of FFA metabolism in the plasma and follicular fluid and determine its effects on oocyte quality in women with PCOS undergoing IVF.

In PCOS, the defective glucose metabolism observed at the systemic level can also be expected at the follicle and ovary level, which may be reflected in the follicular fluid composition. Based on the research of hyperinsulism, it has been postulated that reduced availability of glucose in the oocytes and follicular cells may be caused by defective transportation of glucose can provoke various alternative energy pathways, which would then result in altered levels of various biomolecules in the follicular fluid, such as ketone bodies, lipids, and amino acids (25).

Our findings clearly show that PCOS is associated with significantly higher FAI and IR levels and decreased plasma SHBG levels, independent of BMI. In the subgroup analysis, obese women with PCOS had higher total T and fasting insulin levels than both the control subjects and nonobese women with PCOS. IR is a common but not universal feature of PCOS, and it is not always associated with an increased BMI. Indeed, many studies have shown that both lean and obese women with PCOS have IR (26), although recent studies have shown that there is an intrinsic IR in PCOS that worsens with increasing BMI (27). Hyperandrogenemia may be a predictor of obesity and IR in PCOS patients (28–30). SHBG binds T and dihydrotestosterone with high affinity, and the resultant changes in the SHBG concentrations lead to altered androgen and estrogen delivery to target tissues. However, SHBG blood levels are also regulated by a series of other factors, including estrogens, iodothyronines, and GH as well as stimulating agents and androgens and insulin, which serve as inhibiting factors (31).

In terms of lipid metabolism, obese women with PCOS had higher triglyceride and LDL levels and a lower HDL level than the control group; these changes were manifested as lipid disorders and dyslipidemia in the women with PCOS. Our results are partly in line with the findings of other investigators who evaluated the effect of obesity and PCOS on glucose, lipid, and insulin metabolism (32–34). Morin-Papunen et al (35) found significant metabolic differences between obese women with and without PCOS but not between lean women with and without PCOS. According to Gambineri et al (34), although PCOS per se may be associated with alterations of both lipid and lipoprotein metabolism, the coexistence of obesity usually leads to a more atherogenetic lipoprotein pattern. A greater reduction of HDLs together with a higher increase in both triglyceride and total cholesterol levels has been observed in obese women with PCOS in comparison with nonobese women with PCOS.

Our findings demonstrated that oleic, palmitic, stearic, undecanoic, nutmeg bean, and linoleic acids are the predominant FFAs in the plasma and ovarian follicles. Although linoleic acid is an essential FFA obtained from the diet, the others are synthesized by the body. The predominant FFAs in the ovarian follicles were consistent with those found in the plasma, and the FFA concentrations in the follicular fluid were found to be positively correlated with the FFA concentrations in the plasma (Tables 2 and 3). The conditions that affect FFA concentrations and composition within the plasma and ovarian follicular fluid are not clear. It was postulated that insulin plays a central role in the regulation of lipid oxidation, mainly by inhibiting the release of FFAs from fatty tissue. In insulin-resistant subjects, this inhibition is compromised, leading to an increased concentration of FFA in the bloodstream. The increased levels of circulating FFAs may further contribute to the IR associated with both obesity and PCOS (26) and to the association of PCOS and obesity with the metabolic syndrome and nonalcoholic fatty liver disease (36). Higher FFA concentrations and a defective suppression of the rate of lipid oxidation have been found during hyperinsulinemic clamp in PCOS subjects (35, 37).

In our study, the metabolic profiles of both plasma and follicular fluid indicated the dramatically increased levels of two long-chain fatty acids (palmitic acid and oleic acid) in PCOS patients compared with the control subjects, irrespective of obesity. Moreover, we also found higher concentrations of linoleic acid in the follicular fluid (but not in the plasma) of obese PCOS patients than in that of the control subjects and nonobese PCOS patients. These results suggest that cells of the ovarian follicle metabolize specific FFAs at different rates or perhaps that certain FFAs are transported preferentially into the ovarian follicle. In line with our findings, several published studies have reported increased levels of plasma long-chain fatty acids such as linoleic and oleic acids as well as increased levels of palmitoleic acid in obese women irrespective of PCOS, suggesting that increased lipolysis was possibly secondary to impaired insulin action in adipose tissue (38, 39).

It has been suggested that neutral lipids in oocytes fulfill an important function in supplying energy and in the biosynthesis of membranes during early embryonic development (20, 40, 41). Thus, altered physiology related to energy metabolism may play a role in poorer pregnancy outcomes in women with abnormal lipid metabolism receiving IVF. Other studies have reported that the fatty acid content of follicular fluid in women is associated with poor cumulus-oocyte complex (COC) morphology (17) and that treatment of bovine COCs with increasing doses of specific fatty acids impairs oocyte maturation and subsequent embryo development (21, 42, 43). There is evidence that an elevated BMI is associated with changes in the preovulatory follicular fluid environment, including increased levels of insulin, triglycerides, and androgens (44) and decreased levels of human chorionic gonadotropin (45). Indeed, in our patient cohort, the level of total FFAs in the follicular fluid was linked with the triglyceride levels (r = 0.15; *P* = .04) and IR calculated using HOMA-IR (r = 0.45; *P* = .02 by Spearman correlation). In the IVF cycle, associations between elevations in total follicular FFAs and poorer COC quality have been found, suggesting that excess FFAs adversely influence ovarian follicular function (46). When mouse oocytes were exposed to follicular fluids of differing lipid contents in vitro, maturation of COCs in a lipid-rich environment promoted lipid accumulation and increased the mRNA expression of the lipid droplet protein in mouse COCs (47). It was postulated that this disrupted physiological maturation environment is detrimental to oocytes, leading to the induction of multiple endoplasmic reticulum stress markers and impaired maturation.

Some published studies has discovered that the changes in the fatty acid content of follicular fluid will affect oocyte quality, possibly by influencing its lipid metabolism (17, 21, 48). In line with these reports, our results revealed that the postfertilization developmental competence of oocytes is associated with some specific FFAs in follicular fluid. In PCOS patients, the fragmentation score of day 3 embryos was positively correlated with the oleic acid concentration within follicular fluid, independent of obesity. In addition, the blastomere score was negatively correlated with the stearic acid concentration in the obese PCOS patients. According to the research of Mu et al (49), saturated FFAs such as palmitic acid (C16:0) and stearic acid (C18:0) could induce cell death via apoptosis in human ovarian granulosa cells. Furthermore, in vitro studies have found that unfertilized and immature oocytes are both capable of incorporating fatty acids into their neutral lipid and phospholipid fractions. Palmitic and stearic acids decreased lipid storage and reduced postfertilization developmental competence (49, 50). Therefore, it is possible that apart from the indirect effects on granulosa and cumulus cells, exposure to different FFAs could directly influence the oocytes. Oleic acid, which is mainly present in the plasma and follicular fluid, was reported to have no adverse effects at high doses, but it caused a slight increase in lipid storage and postfertilization development (46). Oleic acid was also capable of compensating for the adverse effects of palmitic and stearic acid. This implies that not only the concentrations but also the ratio of saturated and unsaturated fatty acids in the follicular fluid affect the developmental competence of oocytes.

In conclusion, spectroscopy-based analysis revealed that in addition to decreased insulin sensitivity and abnormal lipid metabolism, specific FFA concentrations within the plasma and follicular fluid resulted in different characteristics between the control and PCOS groups. Furthermore, obese PCOS patients had higher palmitoleic acid and oleic acid levels in both the plasma and follicular fluid than did the controls and nonobese PCOS patients. Linoleic acid (C18:2n6cis) concentration was increased in the plasma but not in the follicular fluid of obese PCOS patients as compared with the concentrations in the other groups. Importantly, our results indicated that oocyte developmental competence is associated with oleic and stearic acid concentrations, which may contribute to the mechanisms of poor pregnancy outcomes in patients with PCOS. We propose that further studies with a larger number of patients be conducted to validate our study's findings and to identify novel treatments that may improve fertility in these affected women.
